# Chicken Manure as a Sustainable Bile Acid Source for Biotechnological Applications

**DOI:** 10.1111/1751-7915.70178

**Published:** 2025-06-08

**Authors:** Johannes Holert, Rudolf Wilhelm, Jens Henker, Claudia A. Reinker, Franziska M. Müller, Bodo Philipp

**Affiliations:** ^1^ Institute for Molecular Microbiology and Biotechnology, Microbial Biotechnology & Ecology Group University of Münster Münster Germany; ^2^ Dr. Falk Pharma GmbH Freiburg Germany; ^3^ Environmental Microbiology Fraunhofer Institute for Molecular Biology and Applied Ecology Schmallenberg Germany

**Keywords:** bile acids, CDCA, chicken manure, pseudomonas putida, steroids, UDCA, valorisation

## Abstract

Ursodeoxycholic acid (UDCA) is widely administered to dissolve gallstones, treat liver disorders and reduce blood cholesterol levels. This study investigated fresh and dried chicken manure as a sustainable bioresource for chenodeoxycholic acid (CDCA), a precursor for the biotechnological production of UDCA. For this, bile acids from five commercial dried and seven fresh chicken manure samples were analysed. The bile acid pool consisted predominantly of CDCA (30%–90%) and 7‐keto lithocholic acid (7k‐LCA, 8%–56%), with minor amounts of cholic acid. CDCA concentrations varied between 62 and 2990 mg per kg dry weight, and the highest concentrations were found in two samples from fresh chicken manure, confirming that chickens can produce high but varying amounts of faecal CDCA. As a proof of principle, a newly created *Pseudomonas putida* KT2440 strain expressing a heterologous 7α−/7β‐hydroxysteroid dehydrogenase system was shown to be able to transform manure‐derived CDCA into UDCA without prior substrate purification from raw ethanolic chicken manure extracts. These results demonstrate that chicken manure can be used as an untapped resource for bile acids for biotechnological applications, providing a novel approach for the valorisation of this bioresource.

## Graphical abstract

This study highlights that chicken manure represents an abundant but untapped resource for bile acids, which are in high demand for pharmaceutical and biotechnological applications. We further show that the predominant bile acid, chenodeoxycholic acid, can be used directly from ethanolic manure extracts to produce pharmaceutically relevant ursodeoxycholic acid.

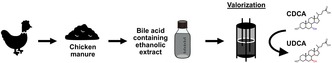

## 1 Introduction

**FIGURE 1:**
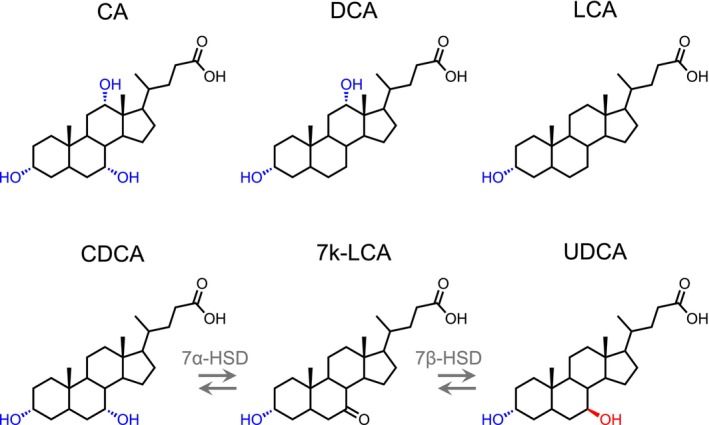
Two‐step biological transformation of chenodeoxycholic acid (CDCA) into ursodeoxycholic acid (UDCA) via 7‐keto lithocholic acid (7 k‐LCA) catalysed by 7α‐ and 7β‐hydroxysteroid dehydrogenases (HSD). Hydroxyl groups in α orientation are blue, in β orientation red.

Bile acids are surface‐active steroid compounds, which play critical roles in nutrient absorption in the digestive tracts of vertebrates (Ridlon and Gaskins 2024). In humans, the two primary bile acids cholic acid (CA) and chenodeoxycholic acid (CDCA, Figure 1) are synthesised in the liver, conjugated to glycine or taurine and concentrated in the gallbladder, before their release into the duodenum. Here they form mixed micelles with dietary lipids and lipid‐soluble vitamins, aiding their absorption for human metabolism. In the terminal ileum, approximately 95% of the bile acids are reabsorbed (Ridlon and Gaskins 2024). The remaining 5% enter the large intestine, where CA and CDCA are converted into the secondary bile acids deoxycholic acid (DCA) and lithocholic acid (LCA) by removal of the 7α‐hydroxyl group by the gut microbiota (Funabashi et al. 2020; Ridlon et al. 2023), which become part of the circulating bile acid pool.

In addition to their function as emulsifying detergents, bile acids function as signalling molecules in diverse metabolic processes by activating complex signalling pathways that regulate bile acid and cholesterol homeostasis, glucose and lipid metabolism, energy homeostasis and inflammation (Mohanty et al. 2024; Ridlon and Gaskins 2024; Vítek and Haluzík 2016; Zhou and Hylemon 2014). Accordingly, bile acids and adverse alterations in their physiology are implicated in a wide range of diseases, including cardiometabolic and neurological disorders, inflammatory bowel disease (IBD) and different cancers (Collins et al. 2023; Fleishman and Kumar 2024). Animal bile has been used for centuries as a natural therapeutic to treat diseases of the digestive, cardiovascular and urinary systems among other disorders (Wang 2014). Today, there is a sizable and growing global market for bile acid pharmaceutics and some individual bile acids such as ursodeoxycholic acid (UDCA, Figure 1) are widely administered for their therapeutic use in bile acid related diseases. UDCA is a natural 7β‐epimer of CDCA, which is produced in humans and most other vertebrates by the intestinal microbiota (Collins et al. 2023) (Figure 1). While UDCA makes up only around 1%–5% of the biliary bile acid pool in most vertebrates, it is the dominant bile acid in some bear species and in nutria, suggesting that UDCA is a primary bile acid in these animals (Hagey et al. 1993; Tint et al. 1986). UDCA is of significant industrial interest as an anticholestatic agent, to treat liver disorders such as primary biliary cholangitis (Beuers et al. 1992; Bowlus et al. 2016), for the dissolution of cholesterol gallstones (Hofmann and Hagey 2014) and for its potential as a chemo‐protecting or chemotherapeutic agent (Goossens and Bailly 2019). Traditional large‐scale industrial production of UDCA is accomplished by different chemical preparation routes from CA or CDCA precursors with comparatively low yields and an adverse environmental impact (Song et al. 2023; Tonin and Arends 2018). Several alternative biotechnological UDCA synthesis strategies via enzymatic epimerisation of the 7α‐hydroxy group of CA or CDCA (Li et al. 2024; Zhang et al. 2019) (Figure 1) or the regio‐ and stereospecific hydroxylation at C7 of LCA using P450 monooxygenases (Grobe et al. 2021) are currently investigated. Regardless of the type of UDCA production, the main sources for obtaining the bile acid precursors are bovine, chicken, duck and goose bile (Song et al. 2023) recovered from slaughterhouses during meat processing. One of the main problems regarding the availability of precursors for UDCA synthesis is the anticipated global upsurge in bile acid demand due to an increase of their use in pharmaceutical applications and their limited supply through the meat industry. In addition, the extraction and purification of bile acids from bile requires a complex multistage process that is costly and harmful to the environment (Tonin and Arends 2018). For these reasons, alternative sources are needed to ensure a stable and sustainable supply of bile acid precursors for the production of UDCA and other bile acid derivatives.

An untapped potential source for bile acid precursor compounds for UDCA production is chicken faeces, which contain CDCA as the main bile acid (Bansal et al. 2020; Porru et al. 2022) in concentrations of up to 18.9 mg g^−1^ dry weight (Tyagi et al. 2007). Chicken manure is produced globally in large amounts as a waste product and is marketed as a fertiliser and a feedstock for biogas production. In this study, we analysed the bile acid profiles of several commercially available dried and fresh chicken manure samples and quantified their CDCA content to assess whether these samples could be used as a CDCA source for the production of UDCA. We then investigated UDCA production using a *Pseudomonas putida* production strain expressing a 7α‐ and 7β‐hydroxysteroid dehydrogenase system for the epimerisation of the 7‐hydroxy group of CDCA (Figure 1) with selected manure extracts.

## 2 Materials and Methods

### 2.1 Pelleted and Fresh Chicken Manure

Pelleted chicken manure was ordered from five different vendors (Supplemental Table S1) and stored at room temperature. Fresh chicken manure was collected into sterile collection tubes (Supplemental Table S2), transported to the lab within 1–7 days and stored at −20°C.

### 2.2 Preparation of Pelleted and Fresh Chicken Manure Samples

Commercial chicken manure pellets were ground with a stainless‐steel impact grinder and sieved through a 0.2 mm plastic sieve. To prepare fresh chicken manure samples, 10 g wet manure was weighed into sterile reaction tubes, mixed thoroughly with 20 mL deionised water before freezing at −80°C. Frozen samples were dried to completeness in a freeze drier (Beta 1–16, Christ, Osterode, Germany) for 65 h, leaving around 28%–38% residual dry weight. Dried fresh chicken manure samples were ground and sieved the same way as commercial chicken manure pellets. All further steps were performed in triplicates for each sample with 0.5–1 g of ground chicken manure pellets per extraction.

To determine bile acid recovery rates, each sample was spiked with 100 μL of a 10 mM hyodeoxycholic acid solution in isopropyl alcohol, and the solvent was evaporated overnight at 60°C. To cleave potential bile acid ester bonds, samples were treated by alkaline saponification before bile acid extraction (Subbiah 1973). For this, 3 mL of a 2.0 M KOH solution in 80% MeOH (v/v) was added, samples were vortexed and incubated in a water bath at 90°C for one hour. Saponified samples were cooled to ambient temperature, and 2.5 mL of 2.5 M HCl was added to lower the pH to around 2.

### 2.3 Organic Extraction From Pelleted and Fresh Chicken Manure to Determine Faecal Bile Acids

Although several methods for bile acid extractions with organic solvents from human faeces and other biological materials are described (Humbert et al. 2012; Shafaei et al. 2021; Tyagi et al. 2007; Zhang et al. 2022), comparative methodological analyses for chicken faeces are still missing. Therefore, we tested three water‐miscible solvents, that is, ethanol (EtOH), isopropyl alcohol (IsoP) and acetonitrile (ACN), and three nonwater‐miscible solvents, that is, ethyl acetate (EtOAc), dichloromethane:methanol ((CH_2_Cl_2_):MeOH (2:1, v:v)); and chloroform:methanol ((CHCl_3_):MeOH (2:1, v:v)), for their bile acid extraction performance using ground, sieved and saponified HGF chicken manure pellets (Supplemental Table S1). A volume of 5 mL of the respective organic solvents was added to each sample, samples were mixed well and incubated for 10–15 min for nonwater‐miscible solvents and overnight for water‐miscible solvents at ambient temperature with constant mixing at 30 rpm in a tabletop shaker. Afterwards, samples were centrifuged at 3200 × g for 2 min. For water‐miscible solvents, the supernatants were directly used for HPLC‐MS analysis; for nonmiscible solvents, the organic phases were collected, the extraction procedure was repeated twice, and the resulting organic phases were combined and dried under a gentle N_2_ flow and heating to 60°C. Dried extracts were resuspended in 2 mL KOH (1 M) and 3 mL ACN before HPLC‐MS analysis. If required, extracts were stored at −20°C. From this analysis, EtOAc was selected as the most suitable solvent for bile acid extractions from all other manure samples (Table S1), which were extracted as described above for water‐miscible solvents.

### 2.4 Ethanolic Bile Acid Extractions for Microbial Growth Cultures

To test chicken manure as a CDCA source in growth cultures, 100 g ground and sieved chicken manure pellets from a commercial source (HGF, Table S1) were extracted with EtOH without saponification. For this, 10 mL HCl (1 N) and 500 mL EtOH were added to the ground powder, the mix was sonicated for 30 min, incubated at 60°C for 30 min and then stirred overnight for 16 h at ambient temperature. The mix was then incubated at 80°C for 4 h with additional ethanol backflow, and the extract was centrifuged at 8000 × g for 10 min. The supernatant liquid was concentrated approximately 7‐fold under a nitrogen stream at 60°C. This extract was added at 5% (v/v) in whole‐cell transformation assays.

### 2.5 High‐Performance Liquid Chromatography and Mass Spectrometry

Bile acids were analysed using a Dionex Ultimate 3000 high‐performance liquid chromatography (HPLC) system (Thermo Fisher Scientific; Waltham, USA), connected to an Amazon speed ion trap mass spectrometer (Bruker; Bremen, Germany) with an electrospray ion source (ESI). HPLC was equipped with a reversed‐phase C_18_ column (Knauer; 150 x 3 mm, Eurosphere II, 100–5 C18). Acidified ammonium‐acetate buffer (10 mM, pH 3.4, 1% v/v formic acid, eluent A) and acetonitrile (eluent B) were used as eluents with a flow rate of 0.3 mL min^−1^. The gradient HPLC method started with 10% eluent B for 2 min, increased to 90% eluent B within 20 min, remaining at 90% eluent B for 1 min and returned to 10% eluent B within 1 min, followed by an equilibration of 5 min. All measurements were performed at a column temperature of 25°C. Alternating ionisation of samples was performed with the following settings: capillary voltage 4000 V, plate offset 500 V, nebuliser pressure 22.5 psi, dry gas flow 8 L min^−1^ and dry gas temperature 200°C. For evaluation of measurements, base peak chromatograms (BPC) or extracted ion chromatograms (EIC) with defined m/z values (+/− 0.5) in negative ion mode were used. For cholate m/z values of 407.2, 453.3 and 815.9, for keto derivatives of cholate m/z values of 405.2, 451.2 and 811.9, for dihydroxy bile acids (HDCA, UCDA, CDCA and DCA) m/z values of 391.2, 437.3 and 783.9, and for keto derivatives of dihydroxy bile acids m/z values of 389.2, 435.3 and 779.93 were used. For calculating CDCA and UDCA concentrations, standard curves were obtained by HPLC‐MS using standard concentrations ranging from 0.01 mM to 0.2 mM. CDCA and UDCA standards were prepared from 10 mM stock solutions in isopropyl alcohol diluted into 0.1 mM NaOH. HDCA recovery rates were calculated as the quotient of recovered moles of HDCA divided by the amount of added moles of HDCA.

### 2.6 Cloning and DNA Manipulation

To produce a UDCA production strain, two adjacent genes encoding 7α‐ and 7β‐HSD enzymes from *Clostridium sardiniense* (*absonum*) DSM599 were cloned into the constitutive expression plasmid pBBR1MCS‐2. These proteins catalyse the NADP^+^‐dependent oxidation of CDCA into 7 k‐LCA and the NADP(H)‐dependent reduction of 7 k‐LCA into UDCA respectively (Ferrandi et al. 2012; Lou et al. 2016) (Figure 1). For this, a fragment containing both genes was amplified from genomic DNA of strain DSM599 using the primer pair Cab_7αβ_fw (TTTTTTGGATCCAGGAGGATTTAGATATGAATTT) and Cab_7αβ_rev (TTTTTTTCTAGAATAACCGCTTAGCTTCATC). The purified PCR product was digested with BamHI and SalI and ligated into the corresponding sites of the pBBR1MCS‐2 vector digested with the same enzymes. The resulting plasmid pBBR1MCS‐2::Cab_7αβ was transformed into *E. coli* ST18, and plasmid‐harbouring strains were selected on lysogeny broth (LB) agar plates containing 50 μg ml^−1^ kanamycin and 50 μg ml^−1^ 5‐aminolevulinic acid. The vector was mobilised into *Pseudomonas putida* KT2440 as described previously (Holert et al. 2016). As a control, the empty pBBR1MCS‐2 was also mobilised into strain KT2440. Plasmid‐harbouring *P. putida* strains were selected on LB‐agar plates containing 50 μg ml^−1^ kanamycin, and correct insertion of the DNA fragment was confirmed by colony PCR using the Cab_7αβ_fw/Cab_7αβ_rev primer pair.

### 2.7 Cultivation of *Pseudomonas putida*

*Pseudomonas putida* KT2440 was selected as a model organism for creating a UDCA production strain because of its accessibility to recombinant DNA technologies, high solvent tolerance and stress resistance, which provides great benefits for industry‐scale biotechnological applications (Ankenbauer et al. 2020; De Lorenzo et al. 2024; Nikel and De Lorenzo 2014). *P. putida* KT2440 strains were maintained on solid mineral medium (Philipp et al. 2006) containing 12 mM succinate or on LB plates containing 1.5% Bacto agar (w/v; BD, Sparks, USA). Strains were transferred onto fresh agar plates every 2 weeks. If required, 50 μg ml^−1^ kanamycin was added to the medium after autoclaving. For *P. putida* starter cultures, test tubes containing 3–5 mL LB medium were seeded with the required strain from agar plates and grown overnight for 10–14 h. To test ethanol tolerance and metabolism, 1%–5% (v/v) sterile filtered (0.2 μm PTFE membrane filters) ethanol was added to sterile 3 mL LB or mineral medium and cultures were inoculated from starter cultures to an optical density at 600 nm (OD_600_) of 0.02 and incubated at 30°C and 200 rpm. Growth was followed by measuring the optical density at 600 nm (OD_600_) with a test tube photometer (Camspec M107, Spectronic Camspec, United Kingdom).

### 2.8 UDCA Production Assays

To test CDCA transformation activity of *P. putida* pBBR1MCS‐2::Cab_7αβ and the empty vector control, 3 mL LB liquid cultures containing 1 mM CDCA were inoculated from starter cultures to an OD_600_ of 0.01–0.02 and incubated at 30°C and 200 rpm. To test UDCA production from chicken manure, triplicate 3 mL cultures containing 5% (v/v) concentrated ethanolic HGF extracts in LB medium or in mineral medium without additional carbon source or with 12 mM succinate, were inoculated from overnight starter cultures of *P. putida* pBBR1MCS‐2::Cab_7αβ to an OD_600_ of 0.01–0.02 and incubated at 30°C and 200 rpm. A single 100 mL upscaled transformation culture was prepared in the same way in LB medium. Samples were withdrawn from all cultures at defined time intervals and analysed by HPLC‐MS.

## 3 Results

### 3.1 Establishment of an Organic Bile Acid Extraction Method From Chicken Manure

**FIGURE 2:**
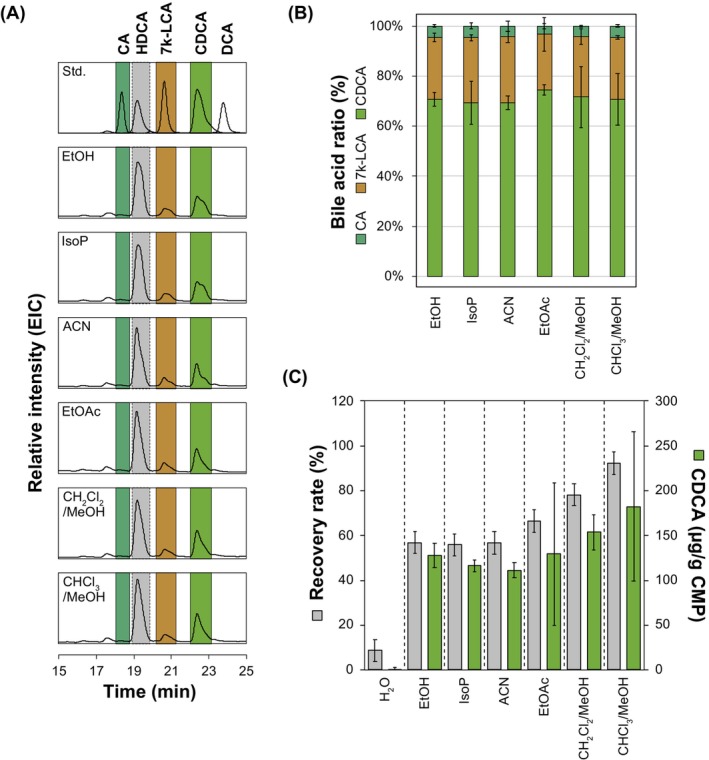
Bile acid extraction from chicken manure pellets with different organic solvents. (A) Representative EIC chromatograms of CA, 7 k‐LCA, CDCA and DCA standards and of organic extracts. (B) Bile acid composition of organic extracts. (C) CDCA yields (green bars) and HDCA recovery rates (grey bars) of organic extracts. Average values and standard deviations of three independent extractions are shown for each sample.

To establish an efficient extraction protocol, we tested six organic solvents for bile acid extraction from chicken manure. For this, commercial chicken manure pellets (HGF, Table S1) were ground and sieved, and bile acids were extracted after saponification using the water‐miscible solvents ethanol (EtOH), isopropyl alcohol (IsoP) and acetonitrile (ACN) and the nonwater‐miscible solvents ethyl acetate (EtOAc), dichloromethane:methanol ((CH_2_Cl_2_):MeOH (2:1, v:v)) and chloroform:methanol ((CHCl_3_):MeOH (2:1, v:v)). HPLC‐MS analysis showed that bile acids were extracted by all solvents (Figure 2A), and MS analyses and comparison to authentic bile acid standards revealed that HGF pellets contained CDCA and 7 k‐LCA as main bile acids and minor amounts of CA (Figure 2A,B). There was little variation in the total bile acid composition between the different solvents, with around 70% CDCA, 25% 7 k‐LCA and 5% CA. While the water‐miscible solvents were able to recover around 56% of the added internal standard HDCA, the recovery rate with nonwater‐miscible solvents was higher, with around 66% for EtOAc, 78% for (CH_2_Cl_2_):MeOH and 92% for (CHCl_3_):MeOH (Figure 2C). Following this trend, the water‐miscible solvents were able to extract around 120 μg CDCA per gram chicken manure, which increased to 182 μg with (CHCl_3_):MeOH. This analysis showed that commercial chicken manure pellets do contain significant amounts of bile acids, especially CDCA and 7 k‐LCA, and that both water‐miscible and nonwater‐miscible solvents can be used to extract these bile acids.

### 3.2 Extraction of Bile Acids From Commercial Chicken Manure Pellets and Fresh Chicken Manure

**FIGURE 3:**
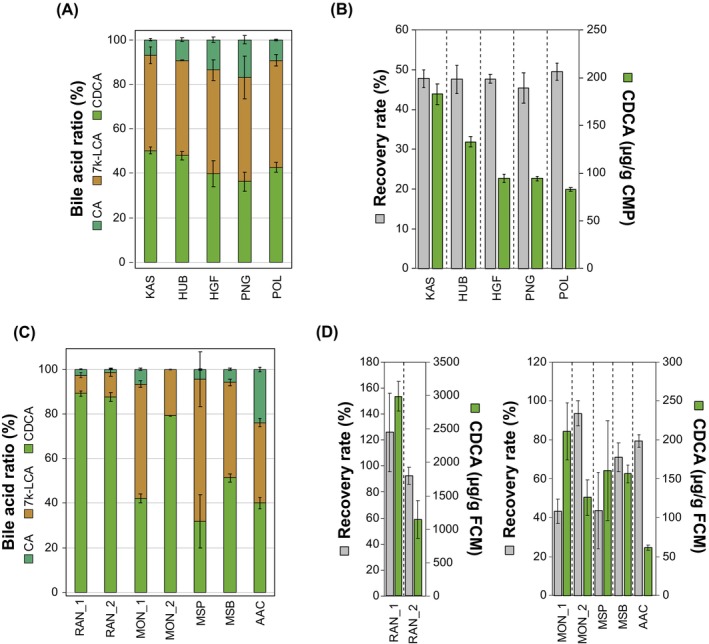
Bile acid extraction from commercial chicken manure pellets and from dried fresh chicken manure with EtOAc. (A) Bile acid composition of five commercial chicken manure pellets. (B) CDCA yields (green bars) and HDCA recovery rates (grey bars) of five commercial chicken manure pellets. (C) Bile acid composition of organic extracts of dried fresh chicken manure. (D) CDCA yields (green bars) and HDCA recovery rates (grey bars) of dried fresh chicken manure. Average values and standard deviations of three independent extractions are shown for each sample.

Based on the high bile acid recovery rate and its lower toxicity compared to chlorinated solvents, we selected ethyl acetate for further bile acid extractions. Bile acids were extracted from five commercial chicken manure pellets (Table S1) and from five fresh chicken manure samples (Supplemental Table S2). Extractions from the commercial samples yielded similar results as in the previous experiment. The bile acid composition was very similar among the samples, with around 40%–50% CDCA, 40%–56% 7 k‐LCA and 5%–10% CA (Figures 3A and S1). Recovery rates were close to 50% for all pellets (Figure 3B), and CDCA yields were between 80 and 180 μg per gram chicken manure.

In comparison, the bile acid content of fresh chicken manure was more variable. Like pelleted manure, fresh manure contained CDCA and 7 k‐LCA as main bile acids and small amounts of CA (Figure 3C, Figure S1). However, the ratio of these bile acids varied strongly among samples. While CDCA made up around 80%–90% of the total bile acid extract in the RAN_1, RAN_2, and MON_2 samples, with around 8%–20% 7 k‐LCA and 0%–2% CA, CDCA was only present at around 30%–50% in the other samples, with 26%–51% 7 k‐LCA and 4%–24% CA (Figure 3C). Similarly, both recovery rates and CDCA yields varied strongly among the extracts. While the samples RAN_1 and RAN_2 contained 2990 ± 222 μg and 1144 ± 284 μg CDCA per gram dry weight, with recovery rates of 126% ± 30% and 92% ± 6%, respectively, the other samples contained significantly less CDCA with around 62–211 μg CDCA per gram dry weight, with recovery rates of 43%–93%.

These results confirm that CDCA is the major bile acid in chicken manure and show that freshly sampled chicken manure can contain significantly higher amounts of CDCA compared to dried chicken manure and other fresh manure samples. However, it is not yet clear what factors influence the amount of CDCA in fresh and dried chicken manure samples.

### 3.3 Establishment of a *Pseudomonas putida* KT2440‐Based UDCA Production Strain

**FIGURE 4:**
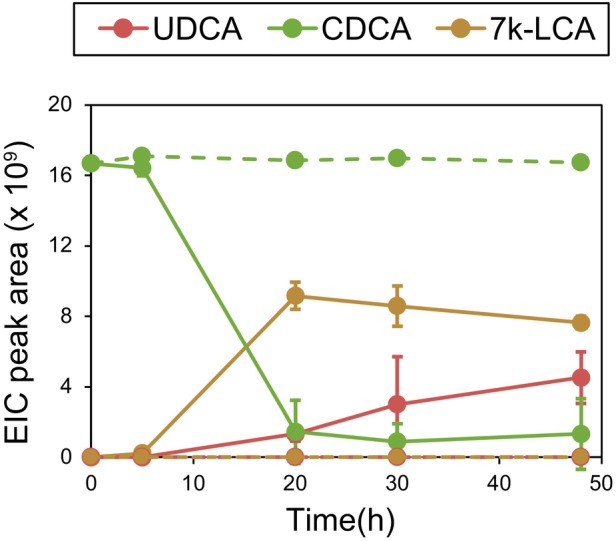
Transformation of CDCA (green) into 7 k‐LCA (brown) and UDCA (red) by *Pseudomonas putida* KT2440 pBBR1MCS‐2::Cab_7αβ (solid lines). No transformation occurred with an empty vector control (dashed lines). Average values and standard deviations of three independent cultures are shown.

To create a model strain for the production of UDCA from CDCA, we cloned a gene cluster encoding a 7α‐ and a 7β‐hydroxysteroid dehydrogenase from *Clostridium sardiniense* DSM599 into the constitutive expression plasmid pBBR1MCS‐2 and transformed the plasmid into *P. putida* KT2440. The resulting strain *P. putida* pBBR1MCS‐2::Cab_7αβ was then tested in batch cultures for its ability to produce UDCA in LB medium with 1 mM CDCA. The strain transformed around 90% of the provided CDCA substrate into 57% 7 k‐LCA and 34% UDCA within 48 h (Figure 4). CDCA was not transformed in control cultures with *P. putida* carrying the empty pBBR1MCS‐2 vector, confirming that the gene cluster from *C. sardiniense* is functionally expressed in *P. putida* for the transformation of CDCA into UDCA.

Next, we tested whether *P. putida* pBBR1MCS‐2::Cab_7αβ can use CDCA from chicken manure as a substrate for the production of UDCA. Since ethyl acetate and other nonwater‐miscible solvents could cause problems in larger scale applications and *P. putida* KT2440 is well known for its ability to tolerate high concentrations of ethanol (Nguyen et al. 2021), we tested ethanolic bile acid extracts of the commercial HGF sample as a CDCA source. Preliminary experiments showed that *P. putida* can tolerate up to 5% ethanol (v/v) in LB medium without significant adverse effects on the growth rate or yield and can grow with up to 2% ethanol (v/v) as sole carbon source in mineral medium (Supplemental Figure S2). Based on these results, we tested CDCA to UDCA transformation activities with ethanolic HGF extracts in small scale batch cultures in LB medium and mineral medium without additional carbon source and with succinate. Similar to the results before, the concentrated ethanolic HGF extract contained CDCA as main bile acid at a concentration of around 360 μM, in addition to 7 k‐LCA as well as small amounts of UDCA. In addition, this extract contained another unknown bile acid with a molecular mass of around 390.14 Da (Supplemental Figure S3), which was tentatively predicted to be 3‐keto chenodeoxycholic acid (3 k‐CDCA).

**FIGURE 5:**
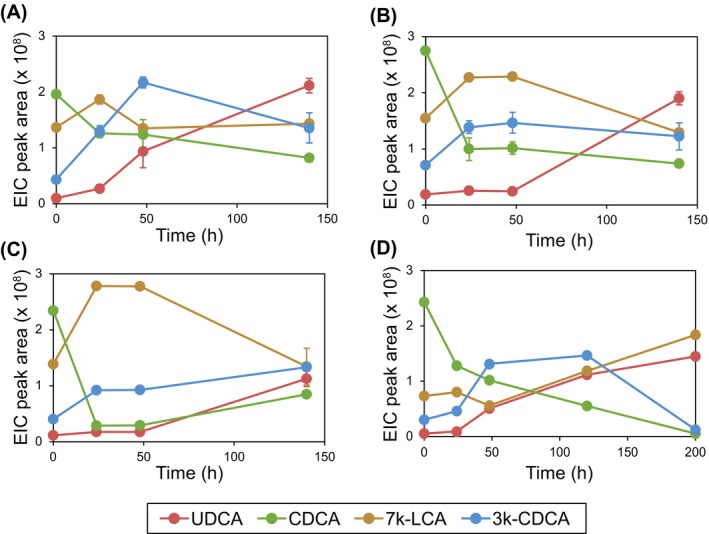
Transformation of CDCA (green) into UDCA (red) by *Pseudomonas putida* KT2440 pBBR1MCS‐2::Cab_7αβ with ethanolic HGF chicken manure extracts also containing 7 k‐LCA (brown) and 3 k‐CDCA (blue) in small scale batch cultures in (A) LB medium, (B) mineral medium with succinate, (C) mineral medium without additional carbon source and in a (D) large‐scale batch culture in LB medium. For (A–C) average values and standard deviations of three independent cultures are shown.

In all tested media, *P. putida* pBBR1MCS‐2::Cab_7αβ was able to produce UDCA (Figure 5A–C), with highest yields of 13.1 + /‐0.8 μM in LB medium, followed by 11.8 + /‐0.7 μM in mineral medium with succinate and 7.0 + /‐0.9 μM in mineral medium without additional carbon source after 140 h of incubation. In LB cultures, CDCA concentrations decreased by around 59% over the course of incubation (Figure 5A), while it decreased by around 73% in mineral medium with succinate (Figure 5B) and by around 88% in mineral medium without additional carbon source (Figure 5C). In the latter cultures, the CDCA concentration increased again towards the end of incubation. While the 7 k‐LCA concentration in LB medium remained constant, it first increased in mineral medium cultures before it returned to values close to the initial concentration. In all cultures, the concentration of 3 k‐CDCA increased during incubation, indicating a 3‐hydroxysteroid dehydrogenase activity of *P. putida* under the tested conditions. While the 3 k‐CDCA concentration decreased towards the end of the incubation in LB cultures, it did not in mineral medium.

In addition to the small‐scale transformation experiments, a single upscaled 100 mL LB culture was also tested for UDCA production with *P. putida* pBBR1MCS‐2::Cab_7αβ. In this culture, UDCA was produced (Figure 5D) with a final yield of around 9 μM. CDCA and 3 k‐CDCA were completely depleted in this culture, while the 7 k‐LCA concentration increased during incubation.

## 4 Discussion

Every year, several million tonnes of chicken manure are produced globally as a waste product from the poultry industry and small‐scale chicken farming, and novel approaches for the valorisation of this renewable resource could contribute to a more sustainable use in a circular economy approach (Gavrilescu et al. 2023). In this study, we show that CDCA and 7 k‐LCA derived from chicken manure can be used as precursors for the biotechnological production of value‐added bile acid products such as UDCA, which provides an untapped opportunity for chicken manure valorisation as a new bile acid resource for biotechnological applications.

Organic extracts of different commercially available dried chicken manure pellets and of fresh chicken manure from different local sources confirmed the findings of other studies that CDCA and 7 k‐LCA are the dominant bile acids in chicken manure (Porru et al. 2022; Tyagi et al. 2007). 7 k‐LCA is presumably produced from CDCA by microbial oxidation of the 7α‐hydroxy group in the large intestine of the chickens (Kisiela et al. 2012) or in the faeces after their excretion, suggesting that both the chicken gut microbiome and the manure storage conditions influence the degree of 7α‐oxidation.

We found CDCA yields of around 1%–45% of the average CDCA yield described for fresh chicken manure in the literature (Tyagi et al. 2007), suggesting that the CDCA content of chicken manure varies significantly between samples, depending on yet unknown factors, which include chicken breed, feed and habitation. The highest CDCA yields in this study were found in two manure samples collected from chickens of three different breeds raised in a private garden. These chickens mainly consumed food that they could find in their habitat, such as grass, earth worms and insects and were additionally fed with kitchen waste, corn and wheat. In comparison, most chickens in our study with lower faecal bile acid concentrations were predominantly fed grain mixtures, which can also be assumed as the main food source for the chickens whose faeces were used to produce the commercial manure pellets tested in our study. It has been shown that diet can influence the amount of intestinal bile acids in chickens and that feeds high in carbohydrates decreased the total bile acid content (Razdan et al. 1997), while a cholesterol‐rich diet increased the total bile acid content (Yang et al. 2024). Chicken breeds with higher intramuscular fat have increased bile acid content (Long et al. 2024), suggesting that broiler breeds might be better suited as bile acid sources than layer breeds. In addition, some bacteria in chicken microbiomes, such as *Alistipes*, correlated with increased serum bile acid contents, suggesting that certain microbes can promote bile acid content also in the faeces (Long et al. 2024). All these factors should be considered in future upscaling scenarios for the use of chicken manure as a useful and cost‐effective bile acid source.

Up to now, most CDCA is extracted from chicken bile derived from slaughterhouses with relatively high yields of about 30% (w/w) CDCA in a complex extraction process requiring significant amounts of organic solvents (Hu et al. 2018; Tonin and Arends 2018). Due to its increasing use as a CDCA source, the price for chicken bile has risen significantly in recent years, and additional CDCA sources are required to meet the growing CDCA market needs. Since chicken manure is produced globally in vast amounts and is mostly marketed as a fertiliser and feedstock for biogas production, this abundant and cheap commodity could represent an additional and economically viable source for bile acids. Assuming that the global broiler chicken manure production exceeds 1.2 million tonnes per day (Chávez‐Fuentes et al. 2017) and that pelleted chicken manure retains roughly 20%–30% dry weight, a theoretical yield of 24–36 tonnes of CDCA could be produced daily even from low faecal bile acid manure containing around 100 mg CDCA per kg dry weight. Given that CDCA is the primary bile acid in all *Galliformes* birds (Hofmann et al. 2010), this can even be extended to other abundant poultry manure samples.

Our results indicate that fresh chicken manure is a better bile acid source than pelleted manure. Fresh manure can be easily collected from poultry houses using automated belt systems or manure scrapers, or by regular manure collection from the roosting area of free‐range operations. After removal of foreign material and larger particles using processes that are already in place for large scale manure pelleting operations, the manure can be ground into a more uniform material before extraction. Although extraction from wet manure should be preferred due to increased bile acid recovery rates (Shafaei et al. 2021), drying and pelleting might be required to lower pathogen loads depending on local regulations. In both cases, the remainder of the manure after bile acid extraction can be further used to extract other valuable nutrients such as nitrogen or phosphorus or for the production of biogas through anaerobic digestion (Dróżdż et al. 2020; Gavrilescu et al. 2023). In addition, removal of bile acids from chicken manure before its use as a fertiliser or a biogas feedstock provides added ecological benefits by preventing the formation of endocrine disrupting compounds by steroid‐degrading bacteria present in soil (Mendelski et al. 2019; Weckerle et al. 2023).

For a biotechnological application of chicken manure as a bile acid source, high bile acid recovery would be desirable. While two extraction steps with chlorinated nonwater‐miscible solvents resulted in higher recovery rates than extractions with water‐miscible solvents, halogenated extractants are undesirable due to health hazards and environmental concerns (Byrne et al. 2016). In contrast, ethyl acetate is considered a greener option but may have negative effects on subsequent biotechnological applications. Given that water‐miscible alcoholic extractants are generally considered to be more environmentally friendly and sustainable (Byrne et al. 2016) and that they are often compatible with downstream applications, they are a better option for large‐scale bile acid extractions. This is especially true for ethanol due to its biocompatibility, low toxicity, bulk availability and low price and because it can be produced from renewable sources and supports circular economy practices (Basile et al. 2018; Rydberg et al. 2004). Accordingly, industry‐scale ethanol‐based extractions are already in use in many applications, including in the food and pharmaceutical industries, which could be adapted for the extraction of poultry manure. However, bile acid recovery from chicken manure using ethanol as an extractant needs to be further improved, for example by performing additional extraction steps, or by using elevated extraction temperatures or coextractants (Zhang et al. 2022). In addition, combination with other techniques, such as solid phase extractions (Zhang et al. 2022) and enzymatic hydrolysis of bile acid conjugates (Dong et al. 2025) could increase bile acid yields and purity. Altogether, sustainable bile acid extraction techniques based on existing methods (Kandrac et al. 2006; Shafaei et al. 2021; Zhang et al. 2022) and the use of green solvents such as ethanol (Byrne et al. 2016) should be applied in upscaled manure extractions to minimise the environmental impact and to meet the strict standards of the pharmaceutical industry.

A newly created *Pseudomonas putida* KT2440 strain expressing the 7α‐ and 7β‐HSD genes from *Clostridium sardiniense* was able to transform manure‐derived CDCA into UDCA without the requirement of prior purification of CDCA from the raw ethanolic extracts. While the highest UDCA yields were achieved when additional carbon sources such as LB or succinate were provided, *P. putida* also produced significant amounts of UDCA without extra growth substrates. This suggests that ethanol, which has already been identified as a cosubstrate for *Pseudomonas* in other biotechnological applications (Byrne et al. 2016; Ziegler et al. 2023), can play two roles in the production of UDCA from chicken manure: serving as an efficient and sustainable bile acid extractant and simultaneously as a carbon and energy source for *Pseudomonas* during UDCA production. In addition, UV/Vis and MS analyses suggested that a wide range of organic compounds were coextracted (not shown), which might act as additional carbon sources. Their concomitant removal from the incubation medium could constitute an initial clean‐up step for chicken manure‐derived UDCA. Taken together, bile acid solubilisation with ethanol is a promising way to provide bile acids from chicken manure together with organic components from the manure to UDCA production strains such as *P. putida* . However, for efficient large‐scale industrial application, the UDCA production genes should be transferred into strains with increased ethanol tolerance and other streamlined *P. putida* chassis better suited for industrial scale productions than the KT2440 wild type (Calero and Nikel 2019; Weimer et al. 2020).

In all cultures, the transformation of CDCA into UDCA was incomplete, leading to the concomitant release of 7 k‐LCA. This indicates that a lack of reduced NADPH hindered the complete conversion of CDCA into UDCA, which is a known phenomenon in such redox reaction‐based whole‐cell production systems. To use *P. putida* as an economical UDCA producer, increased UDCA yields and purity would be desirable, and further experiments are required to move the reaction equilibrium towards UDCA, for example, by coexpressing NADPH‐regenerating glucose dehydrogenase (GDH) (Sun et al. 2013) or formate dehydrogenase (FDH) (Braun et al. 2012), or by targeted modification of the cofactor specificity (Xie et al. 2024) of the involved proteins. In addition, metabolic burden or plasmid loss could have contributed to a decrease in UDCA production over time. To counteract such effects, UDCA production genes should be integrated into the chromosome of the *P. putida* host for a more stable protein production, and protein expression should be made more targeted and controllable by introducing suitable regulatory elements such as promoters (Martin‐Pascual et al. 2021) to fine‐tune UDCA production. In addition, the original *C. sardiniense* gene sequences should be optimised for maximal protein expression in pseudomonads (Schmidt et al. 2023).

## Author Contributions

**Johannes Holert:** conceptualization, writing – original draft, writing – review and editing, data curation, investigation, formal analysis, visualization, methodology. **Rudolf Wilhelm:** writing – review and editing, conceptualization. **Jens Henker:** writing – review and editing. **Claudia A. Reinker:** writing – review and editing. **Franziska M. Müller:** investigation. **Bodo Philipp:** conceptualization, writing – review and editing, supervision.

## Conflicts of Interest

Johannes Holert received funding from Dr. Falk Pharma GmbH to the University of Münster to conduct the study. Rudolf Wilhelm, Jens Henker and Claudia A. Reinker are employees of Dr. Falk Pharma GmbH.

## Supporting information


FIGURES S1–S3.

## Data Availability

All experimental data of this study are contained within the manuscript. Data that support the findings of this study are available in the supporting information of this article.
