# Supplementary material for: Chicken Manure as a Sustainable Bile Acid Source for Biotechnological Applications
Source: Microb Biotechnol. 2025 Jun 8;18(6):e70178. doi: 10.1111/1751-7915.70178 (PMC12146415; doi:10.1111/1751-7915.70178)
Supplement: Supplementary file 1 — FIGURES S1–S3. [file MBT2-18-e70178-s001.pdf]

Tab. ST1: Overview of dried and pelleted chicken manure samples. Samples were ordered online from five different vendors.

Sample	Product	Manufacturer	Ordered
KAS	KAS Hühnerdung	Kulturen Agri Service Stralsund, Stralsund, Germany	10/2019
HUB	Hubey Geflügeldung	Hubey GmbH, Stuhr, Germany	10/2019
PNG	Geflügeldünger Pellets	Premium Nature Green GmbH, Diedorf-Hausen, Germany	10/2019
POL	Pollina Chicken Manure Composting	Alfenatura, Montavo, Italy	10/2019
HGF	HaGaFe Hühnerdung	HaGaFe GmbH, Saterland-Ramsloh, Germany	05/2019

Tab. ST2: Overview of fresh chicken manure samples. Samples were collected into sterile collection tubes and transported to the lab within 1 – 7 days.

Sample	Chicken breed(s)	Chicken feed	Origin	Sampling
RAN_1	Leghorn, Wyandotte bantam, Königsberger	kitchen waste, corn, wheat, gras, insects, earth worms	private garden farming, Randegg, Germany	10/2019
RAN_2	Leghorn, Wyandotte bantam, Königsberger	kitchen waste, corn, wheat, gras, insects, earth worms	private garden farming, Randegg, Germany	07/2020
MON_1	unknown	unknown	private farming, Schruns, Austria	10/2019
MON_2	unknown	unknown	private farming, Schruns, Austria	07/2020
AAC	unknown	kitchen waste, grain mix	private garden farming, Aachen, Germany	10/2019
MSP	Brahma	grain mix	Zoo, Münster, Germany	08/2019
MSB	Guineafowl	grain mix	Zoo, Münster, Germany	08/2019

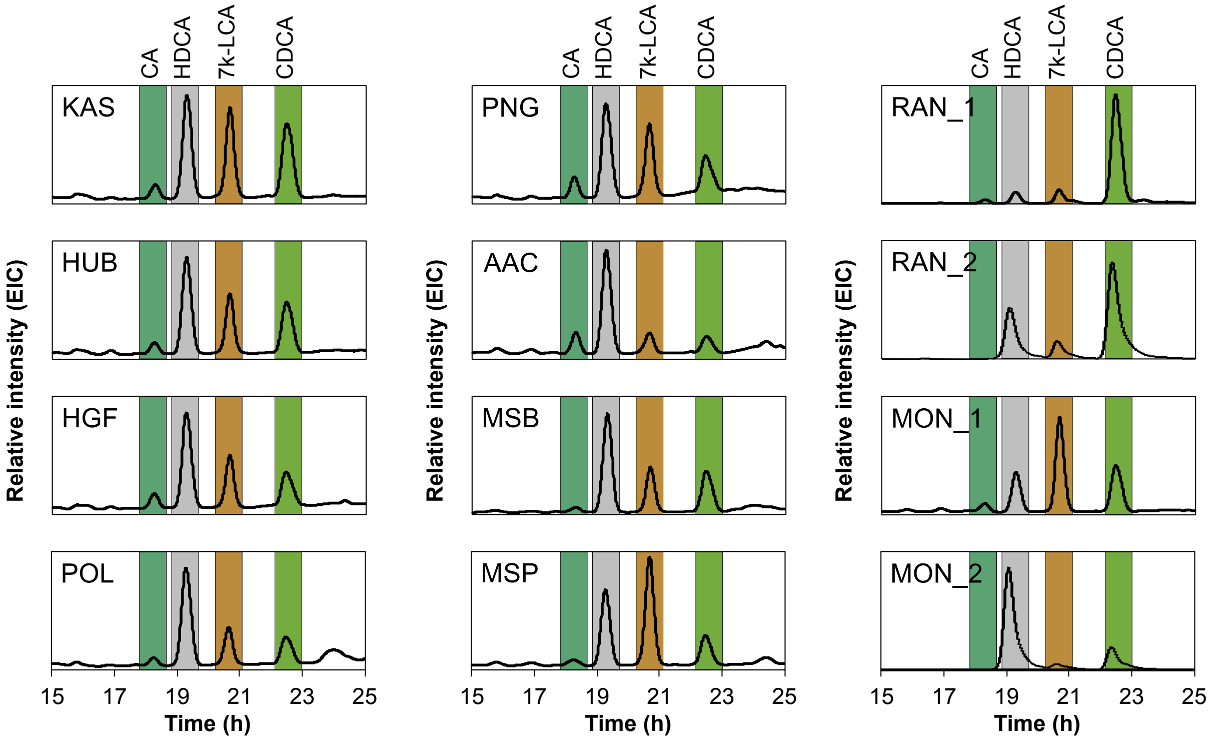

Fig. S1: Representative EIC chromatograms of ethyl acetate extracts from five commercial chicken manure pellets and from five fresh chicken manure samples. Identified bile acids cholic acid (CA), 7-keto lithocholic acid (7k-LCA), and chenodeoxycholic acid (CDCA) and the internal standard hyodeoxycholic acid (HDCA) are labelled. RAN and MON chicken manure samples were extracted twice.

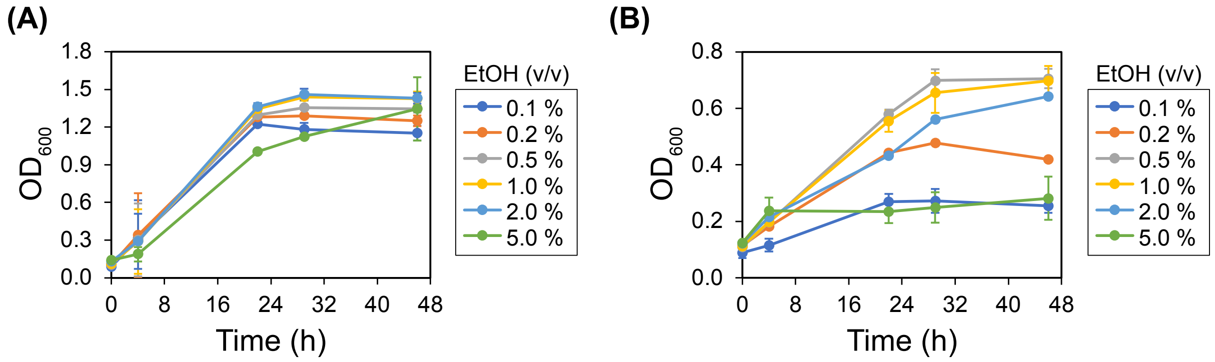

Fig. S2: Growth of *Pseudomonas putida* KT2440 pBBR1MCS-2::Cab_7ab in **(A)** complex LB medium in the presence of up to 5 % (v/v) ethanol and in **(B)** mineral medium containing up to 5 % (v/v) ethanol as only carbon source.

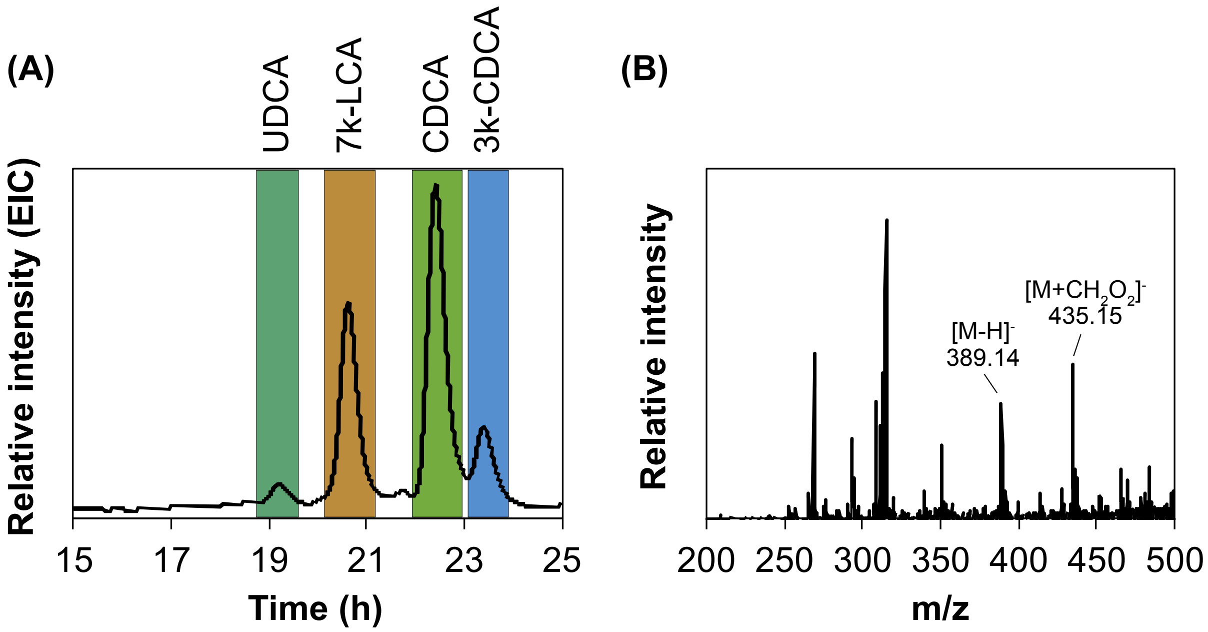

Fig. S3: **(A)** EIC chromatograms of an ethanolic extract from HGF manure pellets. Identified bile acids usrodeoxycholic acid (UDCA), 7-keto lithocholic acid (7k-LCA), and chenodeoxycholic acid (CDCA) are labelled. The extract contained an unknown bile acid with a retention time of around 23.3 min, which was identified as 3-keto chenodeoxycholic acid based on its molecular mass of 390 Da, deduced from its mass spectrum **(B)**.
